# Identification of plasma biomarker candidates in glioblastoma using an antibody-array-based proteomic approach

**DOI:** 10.2478/raon-2014-0014

**Published:** 2014-07-10

**Authors:** Klemen Zupancic, Andrej Blejec, Ana Herman, Matija Veber, Urska Verbovsek, Marjan Korsic, Miomir Knezevic, Primoz Rozman, Tamara Lah Turnsek, Kristina Gruden, Helena Motaln

**Affiliations:** 1 Department of Biotechnology and Systems Biology, National Institute of Biology, Ljubljana, Slovenia; 2 Department of Entomology, National Institute of Biology, Ljubljana, Slovenia; 3 Blood Transfusion Centre, Immunohaematology Department Ljubljana, Slovenia; 4 Educell Ltd., Trzin, Slovenia; 5 Department of Genetic Toxicology and Cancer Biology, National Institute of Biology, Ljubljana, Slovenia; 6 Department of Neurosurgery, University Medical Centre Ljubljana, University of Ljubljana, Ljubljana, Slovenia; 7 Faculty of Chemistry and Chemical Engineering, University of Ljubljana, Ljubljana, Slovenia

**Keywords:** glioblastoma, proteomics, biomarker, antibody array, plasma

## Abstract

**Background:**

Glioblastoma multiforme (GBM) is a brain tumour with a very high patient mortality rate, with a median survival of 47 weeks. This might be improved by the identification of novel diagnostic, prognostic and predictive therapy-response biomarkers, preferentially through the monitoring of the patient blood. The aim of this study was to define the impact of GBM in terms of alterations of the plasma protein levels in these patients.

**Materials and methods.:**

We used a commercially available antibody array that includes 656 antibodies to analyse blood plasma samples from 17 healthy volunteers in comparison with 17 blood plasma samples from patients with GBM.

**Results:**

We identified 11 plasma proteins that are statistically most strongly associated with the presence of GBM. These proteins belong to three functional signalling pathways: T-cell signalling and immune responses; cell adhesion and migration; and cell-cycle control and apoptosis. Thus, we can consider this identified set of proteins as potential diagnostic biomarker candidates for GBM. In addition, a set of 16 plasma proteins were significantly associated with the overall survival of these patients with GBM. Guanine nucleotide binding protein alpha (GNAO1) was associated with both GBM presence and survival of patients with GBM.

**Conclusions:**

Antibody array analysis represents a useful tool for the screening of plasma samples for potential cancer biomarker candidates in small-scale exploratory experiments; however, clinical validation of these candidates requires their further evaluation in a larger study on an independent cohort of patients.

## Introduction

Glioblastoma multiforme (GBM) is the most aggressive primary brain tumour, and following surgical resection, it is conventionally treated with ionising radiation and chemotherapy. The high mortality of patients with GBM is seen by the median survival of 47 weeks, with patients with GBM only rarely surviving more than 3 years.^1^ This is partially due to infiltrative invasion of the single tumour cells into the surrounding parenchyma, where these cells are missed by the treatment strategies that target the bulk tumour mass. Thus, when deciding among adjuvant treatment protocols, there is the need to consider the tumour cellular and genetic heterogeneity, and the development of tumour resistance to such therapies^2^, which are mostly due to the presence of variable numbers of GBM stem-like cells. In the light of this, novel predictive bio-markers of responses to GBM therapy are urgently needed to improve the outcome of GBM therapy, such that these can be monitored pre-operatively and post-operatively in the biological fluids of the patients using a robust and non-invasive method.

Until recently, the search for biomarkers in the body fluids of patients with GBM has not been very extensive^3^, as GBM only rarely shows metastases.^4^ However, recent plasma and serum analyses in glioma patients have been promising, with the identification of some new circulating biomarkers.^5^,^6^ The origin of such secreted molecular bio-markers might be the GBM cells, as these secrete a variety of substances when cultured *in vitro.*^7^ Using proteomic immunological analyses, it has been shown that as well as proteins, cell cultures of brain tumours also secrete protein-loaded microvesicles that can be found in the patient sera.^8^ These biomarkers might also be secreted into the body fluids of patients with GBM by the stromal cells that are a part of the GBM microenvironment. A plethora of cytokines is secreted by the immune cells and mesenchymal stem cells that infiltrate the tumour, which have been reported by us and others to affect GBM aggressiveness, and might thus also be considered as useful biomarkers.^9^ This indicates the importance of the contributions of the tumour micro-environment to the origin of such plasma protein biomarkers. Finally, the systemic host response might result in the release of inflammatory biomarkers, which might also be relevant as biomarkers of glioma progression.^6^,^10^

Different, non-targeted, proteomics approaches have been developed for the identification of molecular biomarkers of glioma progression in patient body fluids, such as in cerebrospinal fluid^11^, ^12^,^13^ and in peripheral blood plasma^14^,^15^ and serum.^16^,^17^ Recently, in the search for novel protein biomarkers, the plasma of patients with GBM was analysed by liquid chromatography/tandem mass spectrometry and compared to healthy volunteers.^15^ Although mass spectrometry is being widely used for cancer biomarker discovery, its use with plasma and serum samples has serious limitations, because of its low sensitivity due to the presence of high-abundance proteins.^18^ Depletion of the most abundant proteins from such samples to allow the detection of putative low-abundance biomarkers might introduce artefacts and bias the quantification.^19^ Therefore, here we propose the use of arrays that are comprised of a broad spectrum of antibodies. In this way, we have quantified the plasma levels of 656 proteins in 17 non-depleted blood-plasma samples from patients with GBM, and compared these with the same protein levels in 17 plasma samples from healthy volunteers. In this study, we identified several plasma biomarker candidate proteins with diagnostic and prognostic potential, which we propose to validate in a larger cohort of patients with GBM.

## Materials and methods

### Ethics statement

This study was approved by the National Ethics Committee and is registered at ClinicalTrials.gov (healthy volunteers: document No. 149/05/08; GBM patients: No. 90/01/11; ClinicalTrials.gov ID: NCT01525459). All of the healthy volunteers and patients with GBM involved signed the informed consent form. Patients were treated in University Clinical Centre Ljubljana, Slovenia.

### Healthy volunteers, glioblastoma patients and plasma sample collection

Seventeen healthy volunteers (HVs) were selected to represent a population distribution considering age, gender (9 female, 8 male) and body mass index, and were analysed in detail in our previous study.^20^ The inclusion criterion for the HVs was age of 20–60 years, and the exclusion criteria were prior record of acute or chronic diseases and pregnancy. None of the HVs had ever been diagnosed with neoplastic disease. All of the HVs were in good health at the time of sampling.

The inclusion criteria for the 17 patients (6 female, 11 male) with GBM (GPs) were: first diagnosis with primary malignant glioma (World Health Organisation WHO grade IV) and age of 20–80 years. The diagnosis was made after surgery, according to standard clinical and histopathological analyses of the tumour tissue at the Neurosurgery Department of the University Clinical Centre in Ljubljana, and the Institute of Pathology of the Medical Faculty. The exclusion criteria for the GPs were pregnancy and brain metastasis.

All of the participants in the study were Caucasians. The HVs and GPs were non-smokers and were not taking oral contraceptives or other drugs for at least 1 month prior to sampling. The morning fasting blood samples were collected between 07:00 and 09:00 hours (on the morning of the surgery for the GPs) from the 17 HVs and 17 GPs. The relevant clinical characteristics of the HVs and GPs are given in the Supplemental Information Table 1.

### Blood sample processing

Eight millilitres of whole blood were drawn from each participant and transferred to preparation tubes containing 1.0 mL 0.1 M sodium citrate (362782, Beckton, Dickinson and Company); these were immediately centrifuged at 1800× *g* for 20 min at room temperature. The plasma was then ali-quoted to separate tubes and stored at −80°C until analysis. All of the blood samples were processed within 1 h of being drawn.

### Antibody arrays

All of the 34 plasma samples from the HVs and GPs were analysed using the Explorer antibody array (Full Moon BioSystems), which has 656 different antibodies spotted on each array, as two replicates.

To clear the plasma samples, 800 μL of each was thawed by centrifugation (20817x g at 4°C for 10 min), as suggested by the manufacturer. Then, 400 μL of the middle clear liquid was transferred into a new tube and analysed using the NanoDrop microvolume spectroscopic technique (Thermo Scientific), to confirm sample clarity and to estimate total protein concentration.

Each sample was analysed on one antibody array slide in five batches, according to the manufacturer specifications. To eliminate any batch effects, each batch was set to consist of an equal number of random HV and GP samples. First, 20 μL of each plasma sample were biotinylated (3 μL of biotin in dimethyl formamide solution, 10 μg/μL, supplied with the antibody arrays), while the antibody array slides were blocked using 3% (w/v) dry milk solution. The coupling step was performed for 2 h, after which the slides were washed intensively 10 times, before being submerged into 60 mL of detection buffer (supplied with the antibody arrays) with 60 μL of Cy3-streptavidin solution, 0.5 mg/mL (GE Healthcare; as suggested by FullMoon BioSystems) for 20 min in the dark; the washing step was then repeated (10 times). After the last wash, the slides were dried by centrifugation (142x g at room temperature for 2 min) and scanned within 12 h using an LS200 microarray scanner (TECAN), with a single-channel laser at 543 nm and the filter at 590 nm, and with 10-μm resolution.

The raw images from the microarray scanner were analysed using ImaGene software (BioDiscovery). Spots of poor quality (*e.g*., unequal signal, artefacts, comet tails) were automatically flagged as poor-quality spots (using the software default settings). The images were also inspected manually, and all of the poor-quality spots were excluded from further analysis (with an “NA” value assigned to them). If the mean signal of the spot did not reach the value of two standard deviations of the background, the spot was flagged as an empty spot. The array values were normalised by quantile normalisation^21^ to correct for any technical, chip-to-chip, or day-to-day variations. As there were two technical replicates of each spot on each microarray, the geometric means between the replicates were calculated. If one of the replicates was flagged as an empty spot or a poor-quality spot, only the other replicate was considered. If the absolute difference between two replicate spots was greater than their geometric mean, the spots were substituted with the “NA” value. All “NA” values were excluded from further statistical analysis. Then all of the spots flagged as empty were substituted by half of the global (over all of the microarrays), minimal, non-empty spot value. This data is available in the Supplemental Information Table 2.

### Statistical analysis of the differences in the plasma protein levels between the healthy volunteers and GBM patients

We used Wilcoxon, Mann-Whitney non-parametric statistical tests with the critical alpha *p* value of 0.05. When all of the GP samples were compared to all of the HV samples, we identified 42 proteins with altered plasma levels.

As there was a significant difference in the mean ages between the HVs and GPs (HV mean age, 39 years; GP mean age, 60 years), we speculated that this difference might account for some of the differences in the levels of these 42 identified proteins. We therefore performed the analysis, where the HVs were separated into the groups of younger HVs (HV_Y_; age, <40 years; n = 8) and older HVs (HV_O_; age, ≥40 years; n = 9), these two HV age groups were compared separately against the GPs (all >40 years; n = 17). We again used Wilcoxon, Mann-Whitney non-parametric statistical tests with the critical alpha *p* value of 0.05, in combination with an absolute log_2_FC >0.5 cut-off, to identify the proteins with altered levels in the plasma samples. We then further analysed only the proteins with significantly altered plasma levels in both the HVs vs. GPs and the HV_O_ vs. GPs comparisons that had absolute log_2_ fold changes >0.5 in both of these comparisons (n = 11).

The list of these 11 biomarker candidates was annotated according to the official gene symbol, UniProt accession ID, tissue expression, molecular class and primary localisation. This annotation was performed using the Human Protein Reference Database (http://www.hprd.org/). We also examined each protein in the Human Proteome Atlas Database (http://www.proteinatlas.org) for the grade of tissue expression in normal tissue and in glial tumours. All of these data are summarised in Table 2. The main functional categories that these proteins correspond to were determined using DAVID Bioinformatics Resources 6.7^22^, to better understand the origins of the differences between the GPs and the HVs.

### Survival analysis of the patients with GBM

The GPs were grouped according to their survival after their diagnosis, to short-term survivors (GP_S_) and long-term survivors (GP_L_). The cut-off for survival was chosen in previous studies as from 6 months^23^ up to 36 months.^24^ To choose a biologically relevant cut-off, we performed Wilcoxon, Mann-Whitney non-parametric statistical tests, using only the cut-offs of 6 months and 12 months survival after diagnosis, as only one of the GPs in the present study survived for more than 24 months. We chose the cut-off of 12 months, as the statistical test revealed more proteins with altered levels in plasma between the GP_S_ and GP_L_ survival groups at this cut-off.

We also determined how well the proteins associated with survival separated between the GPs with different survivals after diagnosis, and determined the antibody array signal value (cut-off value) that provided the most significant separation of the samples. We separated all of the GP samples into two groups according to the cut-off of the antibody array signal. The cut-offs were selected to group samples into two groups (lower and higher signals) in six predetermined ratios: 0.25:0.75, 0.35:0.65, 0.45:0.55, 0.55:045, 0.65:0.35 and 0.75:0.25. The significances of the differences (*p* values) between the groups (for all of the cutoffs) were determined with log-rank analyses. The Kaplan-Meier curves were constructed (R version 2.15.1, libraries KMsurv 0.1–5, knitr 1.2.10, patchD-VI 1.9, survival 2.37–4) to visualise the groups with the most significant differences for each protein.

### Western blotting

The concentrations of the total proteins in the samples were determined using the Bradford assay^25^ 50 μL of 1000×-diluted samples were mixed with 200-μL Roti®-Quant universal reagent (Carl Roth, Karlsruhe, Germany), which was diluted in milli-Q water (1:5) prior to the reaction. The optimal dilution of the samples was calculated to have a final concentration of total protein between 3 mg/ml and 6 mg/ml.

The plasma samples were pooled to have 7 pools for the GPs (4 pools GP_S_, 3 pools GP_L_) and 7 pools for the HVs (3 pools HV_Y_, 4 pools HV_O_) for joint analysis on one transfer membrane. Prior to the loading of each protein sample onto the electrophoresis gel (15 μl), they were diluted 1:50 in phosphate-buffered saline. The electrophoresis of the precast Mini-PROTEAN TGX gradient (4% to 15% acrilamide) gels (#456-1086, BioRad) was performed for 3 h at room temperature and under constant current (25 mA). The separated proteins were transferred from the gel to the immunoblot PVDF membrane (#162-0174, BioRad) at 4°C for 18 h under constant current (45 mA). Two membranes were prepared; one for incubation with the primary anti-GNAO1 rabbit polyclonal antibody (ABIN406520, Antibodies Online; 1:50 dilution), and the other with the primary anti-CDKN1B mouse monoclonal antibody (E6764, Spring Bioscience; 1:100 dilution), at 4°C for 18 h. After washing the membranes with phosphate-buffered saline with 0.1% Tween, they were incubated with the secondary antibodies conjugated with horse-radish peroxidase and diluted 1:2500, for 3 h at room temperature. An anti-rabbit IgG antibody was used for GNAO1 (W4011, Promega), and an anti-mouse IgG antibody was used for CDKN1B (W4021, Promega). After this, the membranes were incubated with Amersham ECL Prime Western Blotting Detection Reagent (RPN2232, Amersham) and exposed to Amersham Hyperfilm MP (#28-9068-42, GE Healthcare), for 5 s. The membranes were washed for 18 h at 4°C, and incubated with a goat anti-human IgM antibody with conjugated horse-radish peroxidase (ABIN102628, Antibodies Online) at a dilution of 1:20,000 for the loading control. The detection proceeded in the same manner as described above.

The Western blotting images were scanned using a standard office scanner at 600 dpi (see Supplemental Information Figure 1 for raw images). The bands were quantified with ImageJ, using the default settings. The data were then normalised according to the IgM levels.^26^ Basic, two-sided, un-paired t-tests were used to determine the *p* values when comparing the different groups; *p* <0.05 was considered as statistically significant.

## Results

### Identification of proteins showing altered plasma levels in GPs compared to HVs, using antibody arrays

To reveal the plasma protein biomarker candidates, we screened blood plasma samples collected from two types of subjects, healthy volunteers (HVs) and GBM patients (GPs), using commercial explorer antibody arrays. To provide a broader overview of the differences in the levels of plasma proteins between the HVs and GPs, Wilcoxon, Mann-Whitney non-parametric statistical tests were performed, comparing the GP samples against the HV samples, with an applied stringency for *p* <0.05. Forty-two proteins with significantly altered plasma levels in the GPs were identified, of which five were increased and 37 were decreased in the GPs (see Supplemental Information Table 3).

**FIGURE 1.**
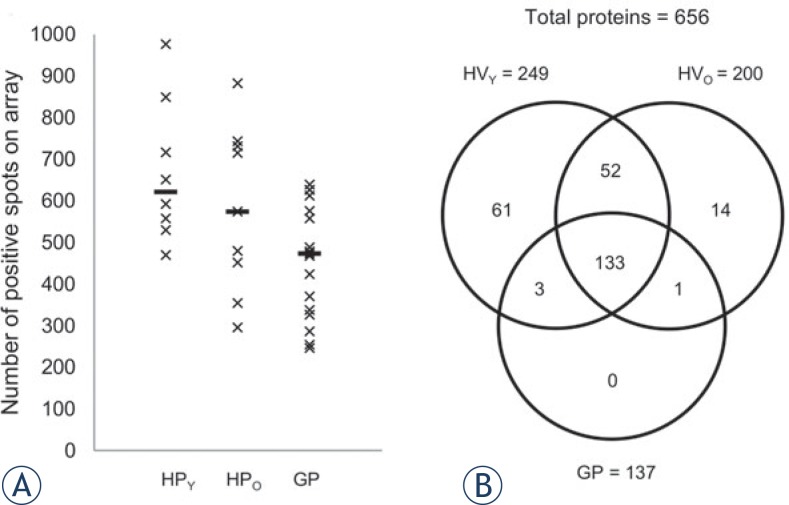
Differences in the detectable levels of the plasma proteins in healthy volunteers and patients with GBM. **(A)** Comparison of the positive spots (of 1312 spots on each array) in the younger HVs (<40 years; n = 8), the older HVs (≥40 years; n = 9) and all of the GPs (>40 years; n = 17). Horizontal bars: median for each group. **(B)** Venn diagram showing the overlap of the detectable proteins in the different patient groups. A protein was considered detectable when it was flagged as positive in ≥75% of the samples in the same group.

In our previous study on HVs^20^, we reported that age did not affect the gene expression in blood cells or the plasma metabolites. In contrast, in the present study, we found that age has a significant impact on the various plasma proteins analysed in the HVs. In addition, fewer low-abundant proteins were detected in the plasma of the older subjects (Figure 1, HV_O_).

To identify the biomarker candidates that were independent of age, we further analysed only the proteins with significantly altered plasma levels in both the HV *versus* GP and the HV_O_ *versus* GP comparisons. These were considered to be the potential protein biomarker candidates (Table 1), of which ferritin (FTL), guanine nucleotide binding protein alpha (GNAO1) and the S100 calcium-binding protein A9 (S100A9) levels were increased; all of the others (FADD, CDKN1B, ICAM1, MLH1, MMP11, POLG, SKP1, ST8SIA1) were decreased.

**FIGURE 2.**
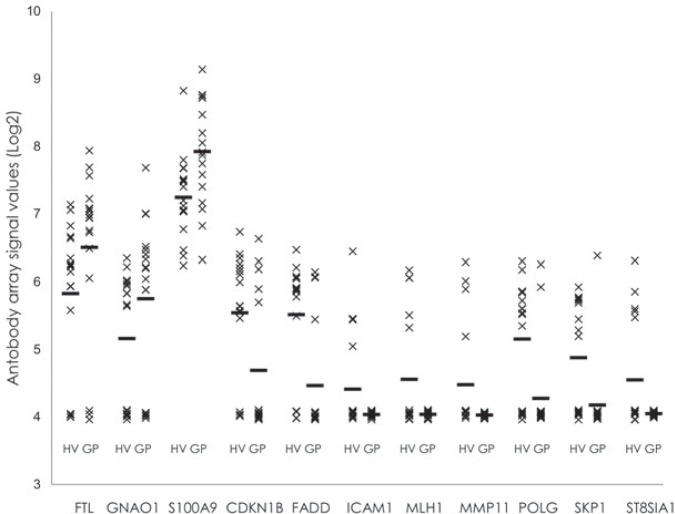
Relative abundances of the putative GBM protein biomarkers in the individual plasma samples analysed. Horizontal bars: mean for each group. A relative protein abundance of 4 represents the background for non-detected proteins.

The distributions of the protein abundance in study participants are presented in Figure 2. ICAM1, MLH1, MMP11 and ST8SIA1 were not detected in any of the GP plasma samples, and were detected in only a limited number of the HV plasma samples. Interestingly, MMP11 (in 4 out of 9 female subjects) and ST8SIA1 (in 5 out of 9 female subjects) were detected only in the HV women (see Supplemental Information Table 2). The biomarker potential of these proteins should be investigated further in a large scale (i.e., with higher sample numbers) clinical trial with a more sensitive assay.

All of the 11 proteins identified as having altered plasma levels were further analysed with the DAVID database to see if they are part of the same processes; three processes were found to be most represented: (1) T-cell signalling and immune responses; (2) cell adhesion and migration; and (3) cell-cycle control and apoptosis. As the group 1 plasma proteins with assigned roles in numerous T-cell signalling and immune response processes were strongly represented, this suggests that at least some of these might have derived from immune cells that responded to the tumour with chemokine paracrine signalling *in vivo*.

### Altered levels of plasma proteins in the GP samples are associated with patients survival

**FIGURE 3.**
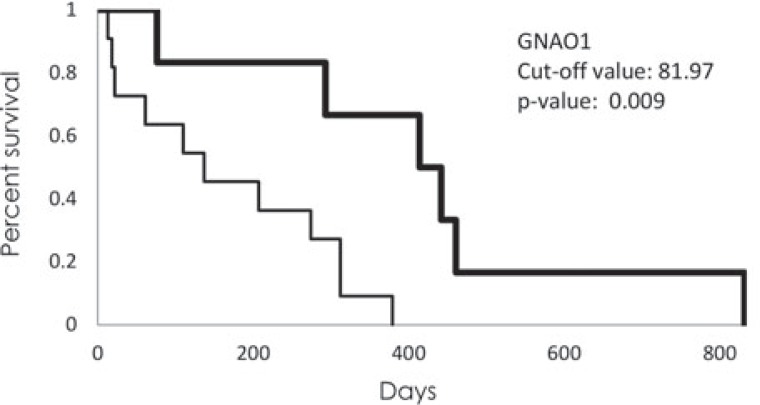
Kaplan-Meier graph of survival of the patients with GBM according to GNAO1 signal intensity. Thick line represents patients with higher signal intensity than cut-off value (81,97); thin line represents patients with lower signal intensity.

To determine whether the survival of the GPs after diagnosis is related to the levels of any plasma proteins, the GP samples were grouped according to the short-term survivors (GP_S_; t <356 days; n = 12) and the long-term survivors (GP_L_; t >365 days; n = 5). As gender had no significant effects on survival (*p* = 0.77), all of the GP samples were analysed as one group. Twenty-three proteins that showed different plasma levels across these groups of patients were identified, using Wilcoxon, Mann-Whitney non-parametric statistical tests, with an applied stringency of *p* <0.05. These 23 proteins were further analysed using log-rank analysis, to determine the antibody array signal values that discriminate best between the patients with short-term and long-term survivals. After this analysis, only 16 proteins remained significantly associated with survival (Table 2), of which five were increased in the plasma of the GP_L_ patients (MYOG, CD8A, GNAO1, ALPL, GHGA), and 11 were decreased (DFFA, MAPR2K1, E2F5, PARP1, CD27, CDC37, TFDP2, STAT1, EIF4EBP1, PDGFA, COL18A1). Kaplan-Meier graphs were constructed for each of these 16 proteins (Supplemental Information Figure 2), of which GNAO1 was the most interesting (Figure 3), as it was associated with GBM presence and survival of GBM patients.

### Western blotting confirmation of CDKN1B and GNAO1

**FIGURE 4.**
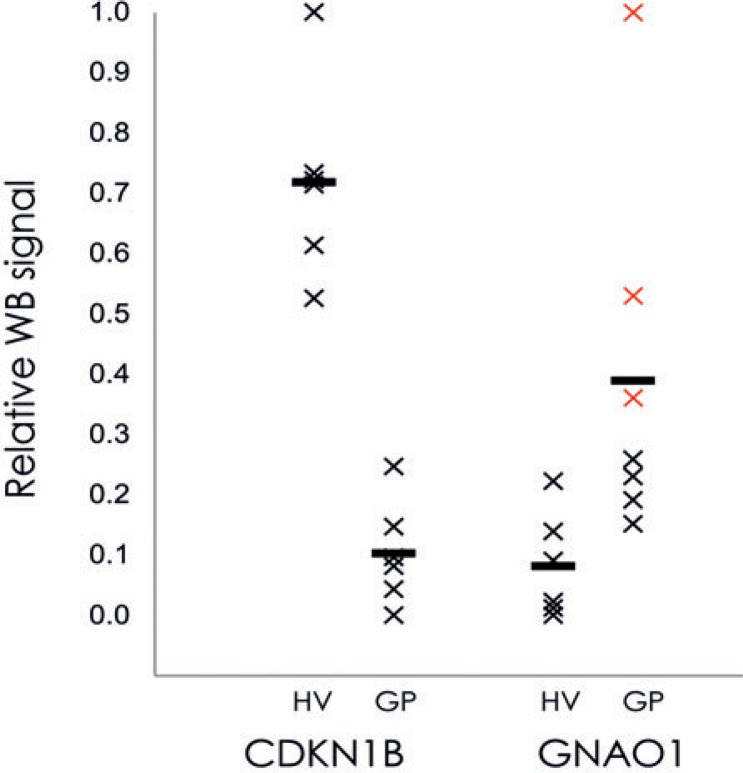
Quantification of Western blotting signals for the two putative plasma biomarkers for patients with GMB: CDKN1B and GANO1. Plasma samples were pooled for the HVs and GPs, and following Western blotting they were quantified and normalized using image densitometry, as described in Material and Methods. Horizontal bars: mean for each group. Red crosses: GNAO1 of three pools of samples from patients who survived longer than 1 year.

To confirm the technical validity of the antibody array method, Western blotting was performed with the pooled plasma protein samples of the HVs (n = 6) and the GPs (n = 6) for the two chosen proteins: guanine nucleotide binding protein alpha (GNAO1) and cyclin-dependent kinase inhibitor 1B (CDKN1B). After quantification of the bands on the raw images (Supplemental Information Figure 1) and normalisation according to the IgM levels, the individual values were plotted according to their relative Western blotting signals (Figure 4). The plasma levels of GNAO1 showed up-regulation of 3.7-fold (*p* = 5.9 e-5) in the GPs compared to the HVs, while CDKN1B showed down-regulation of plasma levels in the GPs, by 5.9-fold (*p* = 8.5 e-8). The plasma levels of GNAO1 were also increased 1.56-fold in the sample pools from the GPs with longer survival compared to sample pools of the GPs with shorter survival (*p* = 0.11). As these data are consistent with the antibody array data, this Western blotting suggests that the antibody arrays provide accurate data as a first step in biomarker identification.

## Discussion

About 90% of human plasma is comprised of the 10 most-abundant soluble proteins.^33^ Although techniques for plasma depletion of these abundant proteins^34^,^35^ have increased the sensitivity of conventional mass spectroscopy methods for proteomic analyses of low-abundance proteins, this depletion is never complete.^34^ Moreover, it has been reported that this procedure for the removal of the abundant proteins might affect the quantitative analysis of low-abundance proteins.^19^ We therefore performed the analyses on the non-depleted plasma samples derived from the HVs and GPs using antibody arrays, through which we have identified novel protein biomarker candidates for diagnosis and prognosis of patients with GBM.

We identified a set of 11 proteins that showed significantly altered plasma levels in the GPs, as compared to the HVs. Using DAVID bioinformatics analyses, we identified three plasma protein clusters that correspond to the following pathways: (1) T-cell signalling and immune responses; (2) cell adhesion and migration; and (3) cell cycle control and apoptosis. These same pathways were reported in one GBM plasma protein study^15^, whereas only the second and the third pathways were identified in a GBM tissue study.^13^ This would imply that the second and the third pathways are involved the intra-tumour cellular cross-talk, whereas the proteins of the first pathway might be involved in host immune system responses to GBM.

Three of the 11 deregulated proteins (FTL, GNAO1, S100A9) were increased in the patients with GBM, where the others were either decreased (CDKN1B, FADD, POLG, SKP1) or were not detected (ICAM1, MLH1, MMP11, ST8SIA1). ST8SIA1 and MMP11 were detected only in the HV women. As ST8SIA1 is involved in breast cancer growth^36^ and MMP11 is mostly expressed in placenta^37^, this would argue for their gender-specific association. Increased FTL and S100A9 have been reported previously for cerebrospinal fluid^11^ and plasma of patients with GP using iTRAQ-based liquid chromatography/tandem mass spectrometry and enzyme-linked immunosorbent assays^15^, thus justifying our methodological approach. Both, FTL and S100A9 are increased in serum under inflammatory conditions, and they might thus represent tumour-related inflammatory responses. The relevance of increased inflammation parameters in the blood of glioma patients, such as erythrocyte sedimentation rate and C-reactive protein levels, has recently been reported^10^, and these parameters might collectively have a strong prognostic significance, as should be tested in further clinical studies, based on the criteria by Gautam *et al.*^15^

Among the proteins CDKN1B (p27/Kip1), ICAM1, MLH1, MMP11 and ST8SIA1, which we found were significantly decreased in the plasma from the patients with GP, only the CDKN1B decrease has been previously reported for plasma by others; the mutation and deregulation of this tumour suppressor protein that is involved in the regulation of cell-cycle progression is a common feature of many cancers^38^, including glioma, where it has been significantly associated with short survival^39^,^40^ and glioma grading^40^ in larger population studies. For POLG and SKP1, which we detected only in the plasma from the HVs, both have been studied previously in GBM tissue^41^,^42^, where POLG expression was associated with mtDNA replication regulator genes, and was interpreted as a prognostic factor.^41^

With respect to prognosis, we found 16 plasma proteins that were significantly associated with GP survival. It is significant that a set of the most strongly associated proteins (*p* <0.01), DFFA, MAP2K1, E2F5, PARP1, predict for longer survival when decreased, which is as would be expected, as these proteins have tumour-promoting characteristics. However, with three proteins, MYOG, CD8A and GNAO1 (*p* <0.01), when these are increased, this predicts for longer survival. In the present study, only GNAO1 - a subunit of heterotrimeric G protein complex was identified as having both diagnostic and prognostic potential. Contrary to our results, in GBM tissue lower levels of GNAO1 as compared to low grade glioma and normal brain tissue have been detected and indicated to affect physical properties of the cell membrane^27^, which may be implying on its secretion from the GBM into the bloodstream. Consistently, we found plasma level of GNAO1 increased 2.9-fold in the GBM patients who showed longer survival, whereas in the GBM patients with shorter survival, it was increased by only 1.2-fold, when compared to the HVs. Although GNAO1 was identified as part of human plasma proteome^43^, it is normally located in the plasma membrane of different cell types. However, mutations in GNAO1 gene can cause abnormal excretion of GNAO1 protein, which was associated with epileptic encephalopathy and disturbed calcium flow in the neuronal cells.^44^ As calcium flow is important for proliferation of neuronal cells^45^, disturbed calcium flow induces apoptosis in GBM cells^46^, which might explain the elevated levels of GNAO1 in GP with longer survival. Although increased plasma levels of GNAO1 have not been associated with GBM to date, this increase has been correlated to poor prognosis in patients with gastric cancer, where high abundance of the GNAO1 protein was suggested to promote cancer-cell viability via pro-apoptotic protein interference.^47^ As we detected lower levels of GNAO1 in the GP_S_ compared to the GP_L_, this coincides with the observation that GNAO1 down-regulation increases proliferation by senescence suppression in hepatocellular carcinoma cells.^48^ Here, we therefore propose that deregulation of this tumour suppressor gene, as reflected by the higher levels of GNAO1 in the plasma of the GPs, might also be associated with prolonged survival of GBM patients.

In conclusion, we have identified novel plasma biomarker candidates that have potential for diagnostic application development. Out of all of the differentially altered plasma proteins in the plasma samples from the patients with GBM, only GNAO1 is predicitve for longer patient survival. To our knowledge, this is also a first association of GNAO1 plasma levels with GBM. We have thus provided evidence that plasma screening using antibody arrays can allow for the identification of novel GBM plasma diagnostic and prognostic biomarker candidates. However, clinical validation of these candidates requires their further evaluation in a larger study on an independent cohort of patients.

## 







## Figures and Tables

**TABLE 1.** Potential plasma protein biomarker candidates for GBM, as identified by the antibody array screening approach used in the present study

**Protein name**	**Gene name**	Difference in protein abundance ^*^	**Molecular class**	**Expression**	**CL**	**ExN**	**ExG**	**Published deregulation**
**Increased in GBM patients**								
Ferritin light chain	FTL	1.65	Storage protein	PL, BC, BR	C	3	1.4	Increased in plasma (MS, ELISA) and in CSF (RIHC) of GPs^11^,^15^
Guanine nucleotide binding protein, alpha	GNAO1	1.65	G protein	BC, BR	M	0	0.4	Decreased in high-grade glioma brain tissue of GPs (IB, IHC)^27^
S100 calcium binding protein A9	S100A9	1.66	Calcium binding protein	PL, BC, BR	C	0	0.3	Increased in plasma of GPs (MS, ELISA)^15^
**Deacreased in GBM patients**								
Cyclin dependent kinase inhibitor 1B	CDKN1B	0.62	Cell cycle protein	BC, BR	N	2	2.0	Decreased in brain tissue in GPs with poorprognosis (IHS)^28^
FAS-associated death domain protein	FADD	0.52	Adapter molecule	BR	C	0	1.1	Up-regulation of TNFR1 through FADD induces apoptosis in GBM cells (RT-PCR, IHS, IB)^29^
Intercellular adhesion molecule 1	ICAM1	0.66	Adhesion molecule	PL, BC, BR	M	0	0.9	Increased in tumour tissue of GPs (RT-PCR)^30^
DNA mismatch repair protein Mlh1	MLH1	0.57	DNA repair protein	PL	N	2	2.4	Decreased in recurrent tumour tissue of GPs (IHS)^31^
Matrix metalloproteinase 11	MMP11	0.62	Metalloprotease	BC, BR	E	0	0.4	Increased in tumour tissue of GPs (RT-PCR, IHS, IB)^32^
DNA polymerase, gamma	POLG	0.54	DNA polymerase	BC	M	2	0.7	/
S phase kinase associated protein 1A (p19A)	SKP1	0.60	Ubiquitin proteasome protein	BR	N	3	2.7	/
Sialyltransferase 8	ST8SIA1	0.59	Sialyltransferase	PL, BC	G	1	1.5	/

* Fold-change in GBM patients when compared to healthy volunteers;

Protein expression: PL = plasma; BC = blood cells; BR = brain;

CL = Cellular localisation; M = plasma membrane; C = cytoplasm; E = extracellular; N = nucleus; G = Golgi apparatus;

ExN; ExG = average grade of protein tissue expression in normal (ExN) and glioma tumour (ExG) tissue, according to the Human Protein Atlas Database (http://www.proteinatlas.org); 0, none; 1, low; 2, medium; 3, high)

MS = mass spectrometry; ELISA = enzyme-linked immunosorbent assay; (R)IHC = (radio) immunohistochemistry; IB = immune-blotting; RT-PCR = real-time polymerase chain reaction

**TABLE 2.** Identification of the 16 proteins that correlate with longer survival of patients with GBM

**Protein name**	**Gene name**	**p value**	Cut-off^*^	**Regulation in longer survivors**
DNA fragmentation factor 45	DFFA	0.002	97.85	Decreased
MEK1	MAP2K1	0.002	64.91	Decreased
E2F transcription factor 5	E2F5	0.003	75.43	Decreased
ADP ribosyltransferase	PARP1	0.006	52.44	Decreased
Myogenic factor 4	MYOG	0.007	108.67	Increased
CD8 Antigen, alpha polypeptide	CD8A	0.009	86.3	Increased
Guanine nucleotide binding protein, alpha	GNAO1	0.009	81.97	Increased
CD27	CD27	0.014	170.66	Decreased
CDC37	CDC37	0.016	312.01	Decreased
Transcription factor DP2	TFDP2	0.018	16.36	Decreased
STAT1	STAT1	0.02	95.77	Decreased
Eukaryotic translation initiation factor 4E binding protein 1	EIF4EBP1	0.023	79.95	Decreased
Platelet-derived growth factor alpha polypeptide	PDGFA	0.03	65.67	Decreased
Alkaline phosphatase, liver	ALPL	0.032	200.13	Increased
Collagen type XVIII alpha 1	COL18A1	0.033	54.68	Decreased
Chromogranin A	CHGA	0.049	80.82	Increased

* Cut-off of microarray signal (intensity), to discriminate patients with shorter *versus* longer survival, as identified for each protein.

## Abbreviations

HV: healthy volunteer;
HV_Y_: HV <40 years old;
HV_O_: HV ≥40 years old;
GP: glioblastoma patient;
GBM: glioblastoma multiforme;
WHO: World Health Organisation
