# Supplementary material for: Identification of plasma biomarker candidates in glioblastoma using an antibody-array-based proteomic approach
Source: Radiol Oncol. 2014 Jul 10;48(3):257–66. doi: 10.2478/raon-2014-0014 (PMC4110082; doi:10.2478/raon-2014-0014)
Supplement: Supplementary file 1 [file 10019-Volume48_Issue_3_06_supp1.pdf]

Sup. Image 1: Western blot scanned images

GNAO1

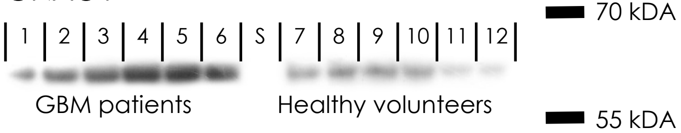

CDKN1B

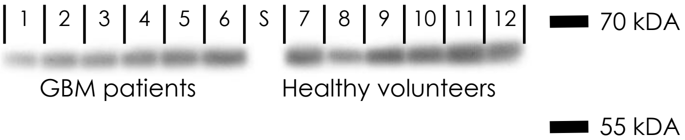

IgM heavy chain

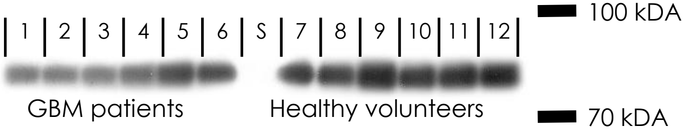

Sample pools:
Pools 1, 2, 3 – GBM patients, short survivors
Pools 4, 5, 6 – GBM patients, long survivors
Pools 7, 8, 9 – Healthy volunteers, age < 40 years
Pools 10, 11, 12 – Healthy volunteers, age > 40 years
S – lane of the standard ladder
