# Supplementary material for: Identification of plasma biomarker candidates in glioblastoma using an antibody-array-based proteomic approach
Source: Radiol Oncol. 2014 Jul 10;48(3):257–66. doi: 10.2478/raon-2014-0014 (PMC4110082; doi:10.2478/raon-2014-0014)

Sup. Image 2: Kaplan-Meier graphs of plasma levels of 16 proteins, associated with survival of GBM patients (C = critical value)

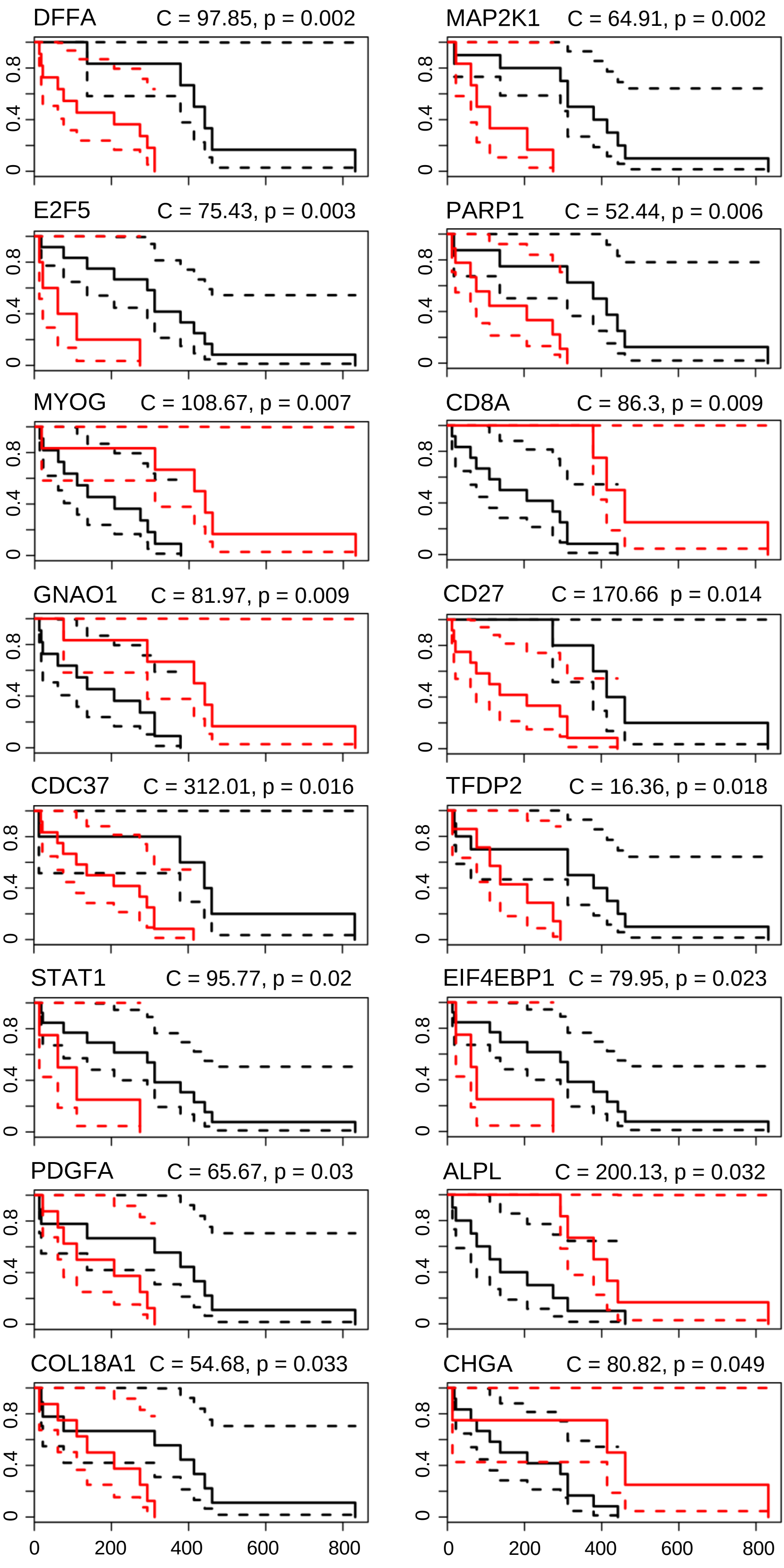

Supplement: Supplementary file 2 [file 10019-Volume48_Issue_3_06_Supp2.pdf]
