# Supplementary material for: Identification of plasma biomarker candidates in glioblastoma using an antibody-array-based proteomic approach
Source: Radiol Oncol. 2014 Jul 10;48(3):257–66. doi: 10.2478/raon-2014-0014 (PMC4110082; doi:10.2478/raon-2014-0014)
Supplement: Supplementary file 3 [file 10019-Volume48_Issue_3_06_Supp3.pdf]

Sup. Table 3: 42 proteins with significantly altered levels in plasma of GBM patients when compared to healthy individuals, determined with two-sided t-test (p-value < 0.05)

Protein Name	Gene Symbol	UniProt Accession	Fold Change (Log2)	Molecular Class	Expression	Primary Localisation
Up-regulated						
S100 Calcium binding protein A9	S100A9	P06702	0,73	Calcium binding protein	PL, BC, BR	C
Guanine nucleotide binding protein, alpha	GNAO1	P09471	0,72	G protein	BC, BR	M
Ferritin light chain	FTL	P02792	0,72	Storage protein	PL, BC, BR	C
Tumor necrosis factor receptor superfamily member 25	TNFRSF25	Q93038	0,38	Cell surface receptor	PL, BC	M
Chromogranin A	CHGA	P10645	0,19	Defense/immunity protein	BC, BR	E
Down-regulated						
Dipeptidyl peptidase IV	DPP4	P27487	-0,31	Protease	PL, BC, BR	M
CD19	CD19	P15391	-0,43	Cell surface receptor	BC	M
Nitric oxide synthase 3	NOS3	P29474	-0,45	Oxidoreductase	PL, BC, BR	M
Thioredoxin reductase 2	TXNRD2	Q9NNW7	-0,46	Reductase	BC, BR	M
Caspase 6	CASP6	P55212	-0,47	Cysteine-type endopeptidase	BC	C
T cell antigen receptor, zeta	CD247	P20963	-0,49	T cell antigen receptor	BC, BR	M
Endoglin	ENG	P17813	-0,49	Cell surface receptor	PL, BC, BR	M
DNA primase	PRIM1	P49642	-0,52	RNA polymerase		N
Interleukin 8	IL8	P10145	-0,54	Cytokine	BC	E
Transferrin receptor	TFRC	P02786	-0,55	Membrane transporter	PL, BC, BR	M
Signal transducer and activator of transcription 5B	STAT5B	P51692	-0,56	Transcription factor	PL, BC	C
E2F Transcription factor 3	E2F3	O00716	-0,56	Transcription factor		N
Tyrosine-protein kinase Lck	LCK	P06239	-0,57	Tyrosine kinase	PL, BC, BR	M
VEGF receptor 1	FLT1	P17948	-0,57	Transmembrane receptor	PL, BC, BR	M
Parathyroid hormone	PTH	P01270	-0,59	Peptide hormone	BC	E
DNA replication licensing factor MCM2	MCM2	P49736	-0,59	DNA binding protein	PL, BC, BR	N
Intercellular adhesion molecule 1	ICAM1	P05362	-0,59	Adhesion molecule	PL, BC, BR	M
GluR delta 2	GRID2	Q4KKU8	-0,62	Membrane transporter	BR	M
Death adaptor molecule RAIDD	CRADD	P78560	-0,62	Adapter molecule	BR	C
Proto-oncogene c-Fos	FOS	P01100	-0,62	Transcription factor	BC, BR	N
DNA replication licensing factor MCM5	MCM5	P33992	-0,65	DNA binding protein	BC, BR	N
Claudin 5	CLDN5	O00501	-0,66	Adhesion molecule	BC, BR	M
Transcription factor AP-1	JUN	P05412	-0,66	Transcription factor	BC	N
DNA dependent protein kinase catalytic subunit	PRKDC	P78527	-0,68	Serine/threonine kinase	PL, BC, BR	N
Junction plakoglobin	JUP	P14923	-0,69	Adhesion molecule	PL	C
Cyclin dependent kinase inhibitor 1B	CDKN1B	P46527	-0,69	Cell cycle protein	BC, BR	N
Matrix metalloproteinase 11	MMP11	P24347	-0,70	Metalloprotease	BC, BR	E
Calcitonin	CALCA	P01258	-0,70	Peptide hormone	BR	E
S phase kinase associated protein 1A (p19A)	SKP1	P63208	-0,74	Ubiquitin proteasome protein	BR	N
Amyloid beta A4 protein	APP	P05067	-0,74	Cell surface receptor	BC, BR	M
DNA repair protein XRCC2	XRCC2	O43543	-0,77	DNA binding protein	BR	N
Sialyltransferase 8	ST8SIA1	Q92185	-0,77	Sialyltransferase	PL, BC	G
DNA mismatch repair protein Mlh1	MLH1	P40692	-0,81	DNA repair protein	PL	N
DNA polymerase, gamma	POLG	P54098	-0,88	DNA polymerase	BC	M
Tyrosine-protein kinase ABL1	ABL1	P00519	-0,95	Tyrosine kinase	BC	C, N
FAS-associated death domain protein	FADD	Q13158	-0,95	Adapter molecule	BR	C
H. Pylori antigens			-1,08	H. Pylori antigens		

PL=Plasma, BC=Blood Cell, BR=Brain

M=Plasma Membrane, C=Cytoplasm, E=Extracellular, N=Nucleus, G=Golgi Apparatus,
